# The impact of osteoporosis on spinal biomechanical stability in adolescent idiopathic scoliosis: a narrative review of finite element analysis and bone mineral density assessment

**DOI:** 10.3389/fbioe.2026.1908609

**Published:** 2026-10-06

**Authors:** Zhaomeng Hou, Jiaojiao Wu, Hua Lu, Chaoqun Chen, Tingliang Han

**Affiliations:** 1 Department of Orthopedics and Traumatology, Yancheng TCM Hospital Affiliated to Nanjing University of Chinese Medicine, Yancheng, China; 2 Department of Orthopedics and Traumatology, Yancheng TCM Hospital, Yancheng, China; 3 Department of Pharmacology, School of Basic Medical Science, Nanjing Medical University, Nanjing, China

**Keywords:** adolescent idiopathic scoliosis, biomechanical stability, bone mineral density, finite element analysis, osteoporosis, scoliosis progression

## Abstract

Adolescent Idiopathic Scoliosis (AIS) is a complex three-dimensional spinal deformity whose precise etiology and pathological mechanisms remain incompletely understood. In recent years, increasing attention has focused on the association between the prevalent phenomenon of reduced bone mineral density (BMD) and osteoporosis in AIS patients and spinal biomechanical stability. This review aims to summarize the epidemiological characteristics and assessment methods of osteoporosis in AIS, with a primary focus on elucidating how it contributes to curve progression by influencing the spine’s mechanical properties. By integrating clinical BMD assessment data with analyses from patient-specific, image-based finite element models of the spine, this article systematically elaborates on the specific mechanisms by which osteoporosis affects the biomechanical stability of the AIS spine. These mechanisms encompass vertebral body strength, spinal flexibility, the risk of curve progression, and the outcomes of internal fixation surgery. This review provides novel perspectives and a theoretical foundation for a deeper understanding of AIS pathophysiology and for optimizing clinical assessment and developing individualized prevention and treatment strategies.

## 1 Introduction

Adolescent idiopathic scoliosis (AIS) is the most common spinal deformity presenting during adolescence, characterized by an abnormal coronal curvature of the spine (Ansari et al., 2024). While it may initially be asymptomatic, AIS can lead to pain, cosmetic deformity, and physical and psychological disability with curve progression (Ansari et al., 2024). As adolescents with AIS transition into adulthood, outcomes vary significantly; some experience curve stabilization, while others face further progression, chronic pain, osteoporosis, fractures, and declines in pulmonary and functional capacity (Ansari et al., 2024). The etiology of AIS is multifactorial, involving a complex interplay of genetic, hormonal, biomechanical, and skeletal metabolic factors (Murray and Bulstrode, 1996). Traditional research has predominantly focused on the morphological alterations of the spine and associated neuromuscular control mechanisms. However, accumulating evidence underscores that AIS patients frequently exhibit a systemic low bone mass state, manifesting as osteopenia or osteoporosis, which may constitute a critical intrinsic factor in the disease’s pathogenesis and progression (Lee et al., 2005a; Hung et al., 2005; Lee et al., 2005b). This systemic bone deficit is not merely a secondary phenomenon but appears to be intricately linked to the fundamental pathophysiology of AIS. For instance, studies have demonstrated that AIS have lower bone mineral density (BMD) at central skeletal sites compared to age- and sex-matched healthy controls (Diarbakerli et al., 2020). Furthermore, this association between low BMD and AIS severity is reinforced by clinical observations showing that female AIS patients with larger Cobb angles exhibit a significantly higher frequency of low BMD (Almomen et al., 2021). The persistence of this low-normal BMD state beyond the peripubertal period and even at peak bone mass in AIS patients, particularly those with more severe curves, suggests a profound and sustained disruption in bone metabolism integral to the disorder (Li et al., 2020).

The biomechanical stability of the spine is a complex construct reliant on the synergistic interaction of bone quality, intervertebral discs, ligaments, and the muscular system. Within this framework, vertebral BMD is a paramount determinant of the spine’s load-bearing capacity and resistance to deformation. Osteoporosis, characterized by diminished bone strength, can critically compromise the spine’s ability to withstand asymmetric mechanical loads, thereby potentially exacerbating the progression of spinal curvature (Zhang et al., 2023). The conceptualization of idiopathic scoliosis as a rotatory decompensation of the spine highlights the importance of spinal stability, which depends on a delicate system of stabilizers where intervertebral disc stiffness is crucial (Castelein et al., 2020). During puberty, rapid increases in spinal loads and moment arms coincide with the maturation of the disc’s anchoring structures, creating a vulnerable period where compromised bone strength due to osteopenia could facilitate mechanical decompensation (Castelein et al., 2020). Finite element analysis (FEA) studies provide direct biomechanical evidence for this relationship, demonstrating that AIS lumbar vertebrae with simulated osteoporosis exhibit increased displacement, reduced stability, and higher stress concentrations under load compared to non-osteoporotic AIS models, especially under extension loads (Zhang et al., 2023). This indicates that osteoporosis not only lowers the threshold for deformation but may actively worsen the existing scoliotic deformity. The mechanical consequences extend to adjacent structures; for example, osteoporotic AIS lumbar spines show greater strain behind the concave side of the intervertebral disc during bending, increasing the risk of disc-related nerve compression (Zhang et al., 2023). Therefore, the interplay between reduced BMD and altered spinal biomechanics forms a potential vicious cycle: initial curvature may induce asymmetric loading, which in the context of low bone quality leads to disproportionate bone loss and further mechanical compromise, driving curve progression.

Advancements in imaging technology have been instrumental in precisely characterizing the bone phenotype in AIS. Dual-energy X-ray absorptiometry (DXA) remains a clinical standard for assessing areal BMD. However, quantitative computed tomography (QCT) offers a three-dimensional, volumetric assessment of BMD, providing superior sensitivity and the ability to evaluate specific vertebral regions. Studies utilizing QCT have consistently revealed significant correlations between lower BMD and greater AIS severity. For instance, a strong negative correlation has been reported between QCT-assessed BMD and both the Cobb angle and vertebral rotation angle in AIS patients (Li et al., 2024). Furthermore, research employing Hounsfield unit (HU) measurements from CT scans has detailed the asymmetric nature of bone loss in AIS, with more pronounced osteopenia typically observed on the convex side of the curve, and the degree of this asymmetry correlating with the severity of the scoliosis. This localized bone quality deficit has direct surgical implications, influencing pedicle screw purchase and the risk of postoperative complications like screw loosening (Cheng et al., 2022). Beyond CT, innovative magnetic resonance imaging (MRI)-based techniques are emerging for opportunistic bone quality assessment. The vertebral bone quality (VBQ) score, derived from T1-weighted MRI signals, has shown utility as a screening tool for low BMD in AIS patients, demonstrating a moderate negative correlation with QCT Z-scores (Yang et al., 2025a). Another study confirmed that patients with AIS have a significantly higher MRI-based VBQ score compared to non-affected controls, indicating lower BMD (Ramos et al., 2022). These imaging modalities collectively enable a comprehensive, multi-faceted evaluation of bone health in AIS, bridging clinical assessment with biomechanical understanding.

Concurrently, the development of personalized three-dimensional finite element (FE) models, constructed from patient-specific CT or MRI data, has revolutionized the *in silico* analysis of spinal biomechanics. These computational tools allow for the simulation and detailed analysis of stress distribution, deformation patterns, and instability risks within the osteoporotic scoliotic spine under physiological loading conditions. For example, a study utilized three-dimensional FE modeling based on CT reconstruction to evaluate vertebral BMD in AIS patients, finding that the overall BMD gradually decreases from the top to the bottom of the spine and that the highest BMD was measured on the concave side of the apex (Han et al., 2023). FEA is extensively applied to optimize treatment strategies. In bracing, FE models help determine optimal three-dimensional corrective forces for different curve types (e.g., Lenke 5) and assess the biomechanical performance of brace designs on pelvic and spinal deformity correction. In surgical planning, FE simulations are used to compare the biomechanical outcomes of different pedicle screw placement strategies, novel correction techniques like Simultaneous Double-Rod Reverse Derotation (SDRRD), and growth-modulation procedures such as anterior vertebral body tethering (VBT). These models can predict postoperative spinal alignment, implant stresses, and the risk of complications like adjacent segment degeneration or screw loosening, particularly in the context of compromised bone quality. The integration of machine learning with FE-generated datasets further enables real-time prediction of postoperative spinal shapes, enhancing preoperative planning efficiency (Phellan Aro et al., 2024). Thus, FEA serves as a powerful translational bridge, connecting the material property of BMD with the macro-mechanical behavior of the entire spinal construct.

This review aims to systematically synthesize the current evidence from two converging research fronts: the clinical assessment of BMD in AIS and the biomechanical insights gleaned from FEA. By integrating findings from DXA, QCT, and MRI-based evaluations with those from computational biomechanical modeling, we will delve into the mechanistic links between osteoporosis and spinal biomechanical stability in AIS. We will explore how systemic and localized bone deficits influence load distribution, spinal flexibility, and deformity progression, and how these factors, in turn, inform conservative and surgical management. The synthesis will provide a comprehensive, evidence-based framework to guide future research directions and refine clinical practices aimed at mitigating curve progression and improving long-term outcomes for individuals with AIS.

## 2 Epidemiology and clinical association of AIS and osteoporosis

### 2.1 Prevalence and characteristics of reduced BMD in AIS patients

A substantial body of evidence from cross-sectional and longitudinal cohort studies consistently demonstrates that AIS exhibit significantly lower BMD compared to their healthy, age-matched peers, with a higher prevalence of osteopenia and osteoporosis (Lee et al., 2005a; Cheng et al., 2000). This systemic reduction in bone mass is not confined to the spine but is a generalized phenomenon. A prospective longitudinal study found that while AIS girls and healthy controls had comparable BMD Z-scores at age 11, the AIS cohort showed a worsening trajectory by age 19, whereas controls improved, indicating a persistent and potentially progressive bone deficit in AIS (Li et al., 2020). This persistence is further corroborated by a surgical cohort study showing that 62.5% of AIS patients with low preoperative BMD at the proximal femur continued to have low BMD even after reaching skeletal maturity, highlighting that bone metabolic abnormalities in AIS are not merely a transient pubertal phenomenon but can extend into adulthood (Nishida et al., 2023). The systemic nature of this bone loss is evident from assessments at multiple skeletal sites. Compared to controls, AIS patients have been shown to have significantly lower DXA-derived BMD values at the lumbar spine (L1-L4), left total hip, left femoral neck, and distal radius (Diarbakerli et al., 2020). Furthermore, studies utilizing opportunistic screening methods, such as MRI-based VBQ scores, have confirmed lower bone quality in the lumbar spine of AIS patients compared to non-affected controls, independent of confounding factors like body weight and height (Ramos et al., 2022). This systemic reduction suggests a fundamental dysregulation in bone metabolism, potentially linked to altered mesenchymal stem cell physiology or endocrine pathways, rather than a localized mechanical effect of the spinal curvature itself (Ko et al., 2020).

Critically, the severity of this BMD reduction is strongly correlated with the magnitude of the spinal deformity, as measured by the Cobb angle. Multiple studies have established a significant negative correlation between BMD Z-scores and Cobb angle, indicating that patients with more severe scoliosis tend to have lower BMD (Li et al., 2024; Shibata et al., 2025). For instance, a study of 348 surgical AIS patients found that those with lower BMD (Z-score ≤ −1) had significantly larger preoperative Cobb angles (59.3°) compared to those with normal BMD (51.4°), and regression analysis identified the BMD Z-score as an independent factor associated with the Cobb angle (Shibata et al., 2025). This relationship is further emphasized by findings showing that female AIS patients with Cobb angles greater than 70° have a markedly higher frequency of low BMD (61.5% lumbar, 69.2% femoral) compared to those with Cobb angles of 40°–70° (14.6% lumbar, 22.0% femoral) (Almomen et al., 2021). The correlation extends beyond global BMD to the local bone environment of the deformed spine. Research utilizing CT HU measurements reveals an asymmetrical loss of vertebral BMD, which aggravates with curve progression. Specifically, vertebral BMD is significantly lower on the convex side of the curve compared to the concave side at periapical levels, and this asymmetry ratio is positively correlated with the major curve Cobb angle (Cheng et al., 2022). Similarly, another study confirmed that patients with lower overall BMD exhibited more severe major curves, and a significant correlation existed between the CT HU values of the apical vertebra (particularly the convex side) and the Cobb angle (Li S. et al., 2025). This localized bone loss pattern may contribute to biomechanical instability and potentially fuel a vicious cycle of curve progression.

The evidence overwhelmingly points to a systemic and persistent bone metabolic disorder in AIS that is intrinsically linked to the pathogenesis and severity of the spinal deformity. The reduction in BMD is prevalent across the axial and appendicular skeleton, persists beyond skeletal maturity, and shows a clear dose-response relationship with curve magnitude. This association underscores the importance of comprehensive bone health assessment in the management of AIS, not only for understanding disease progression but also for mitigating long-term skeletal fragility risks. The integration of advanced assessment tools, such as FE modeling based on three-dimensional reconstruction, offers promising avenues for precise, patient-specific evaluation of vertebral BMD distribution, which could inform surgical planning and biomechanical analyses (Han et al., 2023).

### 2.2 Potential pathophysiological mechanisms by which osteoporosis influences the onset and progression of AIS

The potential pathophysiological mechanisms linking osteoporosis to the onset and progression of AIS are multifactorial, involving a complex interplay of genetic predispositions, hormonal dysregulation, metabolic disturbances, and altered biomechanical loading. Genetic factors play a foundational role, with polymorphisms in genes related to bone metabolism, such as the vitamin D receptor, being implicated in both low BMD and AIS susceptibility (Yang G. K. et al., 2024). Furthermore, Mendelian randomization studies suggest a protective causal effect of higher estimated BMD on AIS risk in females of European ancestry, although this effect appears modified by body mass index (BMI), body fat mass, and vitamin D levels, highlighting the intricate gene-environment interactions (Ghanbari et al., 2023). Hormonal abnormalities are also central to this nexus. Patients with AIS exhibit systemic and persistent low BMD, which is associated with impaired bone mineral metabolism and hormonal pathways (Ko et al., 2020). For instance, the ghrelin pathway, which induces osteoblast differentiation via the GHS-R1a/Gi/cAMP signaling, is impaired in osteoblasts from AIS patients, suggesting a specific cellular mechanism contributing to poor bone formation (Barre et al., 2020). Concurrently, vitamin D deficiency is highly prevalent in AIS populations and is linked to osteopenia, creating a metabolic milieu unfavorable for optimal bone accrual during the critical adolescent growth period (Alsiddiky et al., 2020; Anto et al., 2025). This deficiency, alongside potential disturbances in calcium and phosphorus homeostasis, compromises the material properties of bone. The resultant low BMD fundamentally weakens vertebral bone strength by degrading its microarchitecture—reducing trabecular number, thickness, and connectivity—as evidenced by high-resolution peripheral QCT (HR-pQCT) and DXA studies in AIS cohorts (Diarbakerli et al., 2020; Lam et al., 2021). This microstructural deterioration renders the vertebral bodies more susceptible to microdamage and plastic deformation under the routine mechanical stresses of daily activities.

This compromised biomechanical integrity critically reduces the spine’s “safety margin” for maintaining normal alignment. The vertebral column, with its diminished load-bearing capacity due to osteopenia, becomes less resilient to asymmetric forces. In AIS, the spine is subjected to abnormal, asymmetric mechanical loads due to the existing curvature. FEA demonstrate that osteoporotic AIS lumbar vertebrae exhibit increased displacement and decreased stability under load, with stress becoming more concentrated, indicating a heightened risk of structural failure (Zhang et al., 2023). This biomechanical vulnerability is exacerbated during the adolescent growth spurt, a period of rapid skeletal development and hormonal flux. The combination of low bone strength and the asymmetric pull of paraspinal muscles, along with altered gravitational loading patterns across the scoliotic curve, creates a permissive environment for the initiation and progression of spinal deformity (Skalli et al., 2026). The altered mechanical environment itself can negatively influence bone remodeling in a vicious cycle; regions of the vertebra under abnormal stress may exhibit impaired osteogenic activity, further localizing bone loss. This is supported by findings of asymmetrical osteopenia in AIS, where bone loss is often more pronounced on the convex side of the curve, and the degree of this asymmetry correlates with the severity of the Cobb angle (Cheng et al., 2022; Zhao C. et al., 2024). Therefore, osteoporosis in AIS is not merely a comorbid condition but an integral component of a pathophysiological cascade. It initiates with genetic and endocrine susceptibilities that lead to systemic low BMD and poor bone quality. This compromised bone strength biomechanically destabilizes the spine, lowering its threshold to deform under the asymmetric loads generated by growth and muscle activity, thereby facilitating both the onset and the relentless progression of the scoliotic curvature.

## 3 Application and progress of BMD assessment technology in AIS

### 3.1 Traditional and emerging BMD measurement methods

DXA is the clinical gold standard for assessing areal BMD, commonly measuring sites like the lumbar spine and proximal femur (Nishida et al., 2023; Shibata et al., 2025). Its widespread use in AIS research has consistently demonstrated a significant association between lower BMD and greater curve severity (Almomen et al., 2021; Shibata et al., 2025; Bala and Bala, 2023). Longitudinal studies utilizing DXA have shown that low BMD can persist from the perioperative period through skeletal maturity and even into middle age in AIS patients (Nishida et al., 2023; Akazawa et al., 2024). However, DXA has inherent limitations in the context of spinal deformity. Measurements can be confounded by patient positioning, vertebral rotation, and the superimposition of posterior elements, which are common in scoliotic spines, potentially leading to inaccuracies in the reported areal BMD (Almomen et al., 2021). This is particularly problematic when assessing the lumbar spine, a key site for osteoporosis diagnosis. QCT offers a significant advantage by providing true volumetric BMD measurements and enabling the separate analysis of cortical and trabecular bone compartments (Li et al., 2024). Crucially, QCT is largely unaffected by spinal rotation and deformity, making it a more reliable tool for evaluating local vertebral bone quality in AIS (Li et al., 2024; Yang et al., 2025a). Studies using QCT have confirmed strong negative correlations between volumetric BMD and both the Cobb angle and vertebral rotation angle, reinforcing the link between BMD and deformity severity (Li et al., 2024; Cheng et al., 2022). Furthermore, QCT-based HU measurements allow for the detailed assessment of asymmetrical bone loss across the concavity and convexity of the curve, revealing that BMD is often lower on the convex side and that this asymmetry correlates with the magnitude of the Cobb angle (Cheng et al., 2022; Li S. et al., 2025). It is important to note, however, that while CT HU thresholds are well-validated for diagnosing osteoporosis in adults, normative data and diagnostic thresholds specific to pediatric AIS populations remain an area of ongoing research, requiring cautious interpretation when applied to adolescents. Emerging opportunistic screening methods derived from existing clinical imaging are gaining traction. MRI-based VBQ scoring, particularly using the first sacral (S1) or cervical vertebrae, provides a promising, radiation-free alternative for assessing BMD in AIS patients (Yang et al., 2025a; Yang D. D. et al., 2024; Yang et al., 2025b). The S1 VBQ score shows a moderate negative correlation with QCT Z-scores and demonstrates good diagnostic accuracy for identifying low BMD, offering a valuable tool for pre-surgical screening (Yang et al., 2025a; Yang D. D. et al., 2024). However, it is critical to highlight that current evidence for the VBQ score predominantly rests on studies in adult cohorts. Its validation in pediatric AIS populations is still emerging, and while promising, its generalizability to adolescents with different bone maturation status and growth dynamics requires further investigation. For investigating the fundamental microarchitectural and biomechanical basis of osteoporosis in AIS, HR-pQCT and micro-CT are indispensable. HR-pQCT studies have detailed specific bone quality deficits in AIS, including decreased cortical volumetric density, thickness, and impaired trabecular microarchitecture at critical growth periods like peak height velocity (Zhang J. et al., 2021; Yang et al., 2022; Yang K. G. et al., 2024). Unsupervised machine learning applied to HR-pQCT data has even identified distinct bone microarchitecture phenotypes with differing risks for curve progression, highlighting the prognostic value of deep bone quality assessment (Yang K. G. et al., 2024). These advanced imaging modalities, from QCT to HR-pQCT, move beyond simple density measurements to provide a comprehensive understanding of the structural and material properties of bone, which is essential for elucidating the role of osteoporosis in the pathogenesis and biomechanical stability of the scoliotic spine.

### 3.2 Significance of BMD assessment in the clinical management of AIS

BMD assessment plays a pivotal role in the clinical management of AIS by identifying patients at high risk for osteopenia or osteoporosis, thereby informing comprehensive intervention strategies. A significant body of evidence demonstrates a high prevalence of low BMD in AIS patients compared to healthy controls (Diarbakerli et al., 2020). This systemic reduction in bone mass is not merely a coincidental finding but is intricately linked to the severity of the spinal deformity. Multiple studies have established a significant negative correlation between BMD Z-scores and the magnitude of the Cobb angle, indicating that patients with more severe scoliotic curves tend to have lower BMD (Almomen et al., 2021; Li et al., 2024; Shibata et al., 2025). This association underscores the importance of BMD evaluation as a tool for risk stratification. Identifying AIS patients with low BMD is crucial for implementing targeted non-surgical interventions, such as nutritional supplementation with calcium and vitamin D, and promoting weight-bearing physical activity. Research indicates that vitamin D insufficiency is highly prevalent in AIS surgical candidates (Alsiddiky et al., 2020), and specific genetic interactions, such as those involving WNT16 and vitamin D pathway genes, may influence bone quality and response to supplementation (Yang G. K. et al., 2024). Furthermore, home-based exercise programs have shown feasibility and potential benefits for improving bone health and muscle function in AIS girls (Lau R. et al., 2021; Lau RW. et al., 2021). Therefore, routine BMD assessment, often via DXA, provides an objective basis for clinicians to prescribe these adjunctive therapies aimed at improving skeletal health, which may, in turn, influence the natural history of the condition.

Preoperative BMD evaluation is of paramount importance for predicting surgical outcomes and mitigating complications related to spinal instrumentation. A lower BMD is a significant risk factor for implant-related issues, such as pedicle screw loosening or pullout, due to compromised bone purchase. Advanced imaging techniques, including QCT and MRI-based VBQ scoring, offer detailed assessments of BMD at specific vertebral levels, which is critical for surgical planning (Yang et al., 2025a; Yang D. D. et al., 2024; Yang et al., 2025b). Studies utilizing HU measurements from CT scans have revealed an asymmetrical loss of vertebral BMD in AIS, with greater bone loss on the convex side of the curve, which correlates with curve severity (Cheng et al., 2022). This asymmetry has direct biomechanical implications; the convex side, with lower BMD, presents a higher risk for screw loosening. Consequently, preoperative knowledge of BMD, particularly at the apical and periapical vertebrae, guides surgical strategy. It may influence decisions such as prioritizing distraction on the concave side, using supplemental fixation anchors, selecting larger-diameter screws, or employing cement augmentation on the convex side to enhance fixation strength (Cheng et al., 2022). Furthermore, lower preoperative BMD has been associated with larger preoperative Cobb angles (Shibata et al., 2025; Li S. et al., 2025), which may indirectly relate to surgical complexity. However, it is essential to recognize that the HU thresholds and VBQ cut-offs used for surgical risk assessment are predominantly derived from adult data. Their direct applicability to pediatric AIS patients, who have different bone density profiles and are still accruing skeletal mass, requires ongoing validation. Thus, integrating BMD assessment into the preoperative workup is essential for optimizing surgical planning, implant selection, and ultimately, improving the safety and long-term stability of spinal fusion, with the caveat that interpretation of opportunistic BMD data in AIS must be informed by the current state of pediatric validation.

Longitudinal monitoring of BMD serves as a valuable auxiliary tool for evaluating the effectiveness of non-operative treatments and predicting the risk of curve progression. The persistence of low BMD from adolescence into skeletal maturity and beyond is a well-documented phenomenon in AIS. Longitudinal studies have shown that a substantial proportion of AIS patients with low preoperative BMD continue to have low BMD even after reaching bone maturity (Nishida et al., 2023). Furthermore, research indicates that AIS patients, particularly girls, exhibit a poorer trajectory of BMD accrual during puberty and at peak bone mass compared to healthy controls, with this deficit being associated with greater curve severity (Li et al., 2020). This persistent low bone mass state has long-term implications, as evidenced by studies of middle-aged females who underwent AIS surgery in adolescence, showing significant reductions in BMD and increased prevalence of osteopenia and osteoporosis decades later (Akazawa et al., 2024). In the context of conservative management, such as bracing, serial BMD measurements can provide objective data on skeletal response. For instance, a study on calcium and vitamin D supplementation demonstrated initial bone health improvements after 2 years, but these benefits largely dissipated after supplementation was discontinued, highlighting the potential need for sustained intervention to maintain gains (Lam et al., 2021). Moreover, elevated serum bone turnover markers, such as P1NP, have been associated with a higher risk of curve progression in AIS, suggesting a link between systemic bone metabolism and deformity worsening (Zhang J. et al., 2021). Therefore, tracking BMD changes over time offers clinicians insights into the metabolic bone status of AIS patients, aids in assessing adherence and response to prescribed nutritional and exercise regimens, and provides an additional biomarker that, alongside radiographic parameters, can help forecast the likelihood of scoliosis progression, enabling more personalized and timely clinical decisions.

## 4 The core position and assessment challenges of spinal biomechanical stability in AIS

### 4.1 Definition and components of biomechanical stability

Spinal biomechanical stability is defined as the ability of the spine to maintain its normal alignment under physiological loads, prevent excessive or abnormal deformation, and protect neural structures (Castelein et al., 2020). This stability is not a singular property but emerges from the integrated function of three distinct subsystems: the passive, active, and neural control subsystems, all of which can be aberrant in AIS (Castelein et al., 2020). The passive subsystem comprises the non-contractile structural elements, including the vertebral bodies, facet joints, intervertebral discs, and the ligamentous complex. The integrity of these components, particularly the intervertebral disc, is crucial for spinal stability, as the disc’s stiffness acts as a primary stabilizer (Castelein et al., 2020). The posterior ligamentous complex, consisting of the ligamentum flavum, interspinous, and supraspinous ligaments, is a critical component of this subsystem, contributing 30%–40% of overall spinal stability by limiting biomechanical stress and guiding motion (Anderson and Shahidi, 2023; Kapetanakis et al., 2025). The active subsystem is formed by the surrounding musculature, which dynamically controls posture and movement. The neural control subsystem orchestrates the muscular response through central pattern generators in the spinal cord and sensory feedback from mechanoreceptors in ligaments, discs, and muscles, which is essential for maintaining postural stability and coordinated locomotion (Molkov et al., 2024). Within the passive subsystem, the vertebral body serves as the primary load-bearing structure. Its BMD and strength are foundational to the stability of the passive subsystem, as they determine the vertebra’s capacity to withstand compressive and bending forces without failure (Castelein et al., 2020). This is particularly relevant in the context of AIS, where altered biomechanical loads are distributed across a deformed spinal column. The concept of the functional spinal unit (FSU), comprising two adjacent vertebrae, the intervening disc, facet joints, and associated ligaments, is the smallest structural unit that reflects this integrated stability, responsible for load distribution, shock absorption, and mobility (Navani et al., 2025). Biomechanical stability is thus a dynamic equilibrium. Disruption in any component, such as disc degeneration leading to reduced torsional stiffness, ligamentous laxity, muscular weakness, or impaired proprioceptive feedback, can compromise this equilibrium, leading to instability, deformity progression, and pain (Castelein et al., 2020; Anderson and Shahidi, 2023; St-Georges et al., 2020). In surgical contexts, restoring stability often involves instrumentation that aims to reconstruct this integrated system, with the biomechanical performance of implants—such as pedicle screws, interbody cages, or dynamic stabilization systems—being evaluated based on their ability to restore normal load-sharing and range of motion while minimizing stress on adjacent segments (Jain et al., 2020; Saddiki et al., 2026; Chen R. et al., 2025).

### 4.2 Biomechanical instability mechanisms in AIS

The biomechanical instability in AIS arises from a complex interplay of asymmetric load distribution, structural alterations in vertebrae and intervertebral discs, imbalances in ligamentous tension, and dysfunction of the paraspinal musculature. The unique upright posture of human bipedalism subjects the spine to dorsally directed shear loads, which, when acting on a pre-existing axial plane rotation in a posteriorly inclined sagittal plane during growth, can spark rotational instability (Pizones et al., 2024). This scenario is compounded by factors such as disc maturation, collagen quality, and proprioceptive immaturity, creating the perfect conditions for the development of the three-dimensional deformity (Pizones et al., 2024). Structural changes are fundamental to this instability; AIS patients exhibit significantly more slender vertebrae compared to asymptomatic controls, a trait that is largely independent of curve topology and facilitates mechanical instability (Vergari et al., 2020). This increased slenderness ratio, representing the ratio of vertebral height to transversal size, reduces the structural resistance to bending and rotational forces. Concurrently, altered cervical mechanics, including a loss of cervical lordosis and segmental instability, contribute to abnormal biomechanics associated with cervical buckling, which may be linked to the overall spinal deformity in AIS (Dick, 2025). The paraspinal muscles, crucial for dynamic stabilization, demonstrate impaired function. For instance, the erector spinae muscle on the convex side of the curve shows significantly higher activation during functional tasks compared to the concave side, indicating an asymmetric and compensatory neuromuscular strategy that may not efficiently stabilize the spine (Chan et al., 2023). Furthermore, fatigue of the thoracic erector spinae can lead to significant decreases in translational and rotational mobility on the convex side during certain tasks, highlighting the vulnerability of the system when muscular endurance is compromised (Chan et al., 2023). This muscular dysfunction, combined with the structural predisposition for slenderness and altered alignment, establishes a foundation for mechanical instability that drives the progression of the spinal curvature.

The apical vertebral region, which sustains the greatest bending and rotational stresses, is a critical site for biomechanical failure, particularly when osteoporosis is present. This region is identified as a location of mechanical instability, as suggested by the analysis of vertebral and disc wedging (Vergari et al., 2020). When the vertebrae in this highly stressed zone are affected by osteopenia or osteoporosis—conditions frequently observed in AIS—their diminished BMD and compromised microarchitecture reduce their load-bearing capacity (Han et al., 2023). FE modeling studies have shown that BMD in AIS patients can vary, with the overall spinal BMD gradually decreasing from the cephalad to the caudad regions (Han et al., 2023). In the context of asymmetric loading, this osseous weakness makes the apical vertebrae more susceptible to time-dependent deformation, such as creep, and permanent morphological changes like increased wedging. Computational biomechanical investigations using subject-specific models have explored the stress distribution within the vertebral bone, revealing that the ratio of maximum von Mises stress between the concave and convex sides can be altered by factors like unilateral muscle paralysis, which may further influence local bone remodeling according to Frost’s Mechanostat theory (Hosseinzadeh-Posti et al., 2026). The presence of lower BMD, potentially driven by chronic low-grade inflammatory processes that promote osteoclastogenesis, exacerbates this vulnerability (Kruk et al., 2026). Consequently, the apical vertebra cannot adequately resist the deforming forces, leading to progressive wedging and rotation. This deformation not only worsens the local curvature but also alters the load transmission to adjacent segments, creating a focal point of biomechanical compromise. The interplay between reduced bone quality at the apex and the intense mechanical demands placed upon it establishes a vicious cycle where deformation begets further instability, making this region a primary driver in the escalation of the scoliotic curve.

A self-perpetuating positive feedback loop exists between biomechanical instability and curve progression in AIS: initial instability leads to worsened deformity, which in turn further disrupts load distribution and amplifies local instability. The progression of the three-dimensional deformity alters the normal sagittal and coronal alignments, changing the lines of force acting on the spine. For example, altered dynamic cervical alignment and instability may contribute to the development or progression of the deformity through abnormal buckling mechanics (Dick, 2025). As the curve worsens, the lever arms for muscular forces change, and the asymmetry in load distribution becomes more pronounced. This places abnormal, sustained stresses on spinal components, including the intervertebral discs and ligaments. During crucial periods like puberty, when spinal loads increase rapidly, the integrity of stabilizers like the intervertebral disc is vital; changes in disc properties during maturation can create a window for increased transverse plane vertebral rotation (Castelein et al., 2020). The body attempts to compensate through muscular adaptations, such as the asymmetric activation patterns seen in the multifidus and erector spinae during specific stabilizing exercises (Kim et al., 2026). However, these compensations are often insufficient to restore stability and may even contribute to muscle imbalance and fatigue (Chan et al., 2023). Surgical interventions like posterior spinal fusion aim to break this cycle by providing immediate structural stabilization. Biomechanical computational studies show that surgical correction maneuvers, such as segmental translation and apical vertebral derotation, can significantly reduce Cobb angles and apical vertebral rotation (Wang et al., 2023). Furthermore, the density and pattern of pedicle screws used in instrumentation influence the forces at the bone-screw interface and the efficacy of derotation, with higher apical screw density correlating with better transverse plane correction (Wang et al., 2023; Mallinos et al., 2025). Post-operatively, improvements in postural stability parameters, such as increased time-to-boundary and reduced center of pressure velocity during standing tests, demonstrate that successful correction and fusion can enhance the body’s stability, thereby interrupting the destructive feedback loop (Osuka et al., 2022; Osuka et al., 2024). Thus, the interplay between deformity and instability forms the core of the progressive nature of AIS, underscoring the importance of interventions that address both the structural misalignment and the underlying biomechanical environment.

## 5 FEA: the bridge connecting osteoporosis and biomechanical stability

### 5.1 Basic principles of FEA and its advantages in spinal research

FEA is a computational simulation technique that discretizes complex geometric structures into a multitude of small, interconnected elements to solve for mechanical responses under specified loads and boundary conditions (Manickam and Roy, 2022). This method is fundamentally grounded in numerical approximations of the governing partial differential equations of continuum mechanics, allowing for the prediction of stress, strain, displacement, and stability in intricate anatomical structures like the spine. In the context of AIS research, the power of FEA lies in its ability to construct highly personalized three-dimensional models of the spine. These models are generated from patient-specific imaging data, such as CT or MRI scans, enabling the precise replication of the spine’s unique geometric morphology, material properties—including the critical distribution of BMD—and the complex articulations and ligamentous connections between vertebrae (Han et al., 2023; Li et al., 2026). The process involves segmenting anatomical structures from medical images, converting them into optimized surface and volumetric meshes, and assigning material properties based on literature or advanced techniques like Physics-Informed Neural Networks (PINNs) to enhance accuracy (Ahmadi et al., 2025a; Ahmadi et al., 2025b).

The primary advantage of FEA in spinal biomechanics, particularly for AIS, is its capacity to conduct “virtual experiments” that are ethically or practically impossible to perform *in vivo*. Researchers can non-invasively quantify the biomechanical behavior of the scoliotic spine under various loading scenarios, such as flexion, extension, lateral bending, and axial rotation (Zhang Q. et al., 2021). This includes detailed analysis of stress distribution on vertebral bodies, growth plates, facet joints, and intervertebral discs under different states of BMD (Yahara et al., 2023; Zou et al., 2024). For instance, FEA models have demonstrated that in AIS, asymmetric load transmission significantly increases stress on the concave side of facet joints, leading to subchondral sclerosis and hypertrophy, which may contribute to curve progression (Yahara et al., 2023). Furthermore, FEA is indispensable for predictive modeling and surgical planning. It allows surgeons to preoperatively simulate and compare the biomechanical outcomes of various interventions, such as different posterior fixation methods, interbody fusion techniques, or corrective osteotomies (Saini et al., 2023; Zhao G. et al., 2024; Long et al., 2024). Studies can evaluate parameters like the range of motion (ROM) reduction, stress on implants (e.g., pedicle screws, rods, cages), and the risk of adjacent segment disease, thereby optimizing the choice of fixation length, screw trajectory, and implant material (Farooqi et al., 2024; Tao et al., 2024; Shi A. et al., 2025). The technique also extends to evaluating conservative treatments, such as the biomechanical effectiveness of different orthotic brace designs by simulating corrective forces and predicting immediate in-brace correction and long-term growth modulation (Guy et al., 2024; Guy et al., 2022; Bakhshian Talkhoncheh and Beigzadeh, 2026). By integrating with musculoskeletal models and advanced computational frameworks, FEA provides a comprehensive, patient-specific platform that enhances our understanding of AIS pathogenesis, supports the development of personalized treatment strategies, and ultimately aims to improve clinical outcomes by breaking the biomechanical vicious cycle of deformity progression (Kamal and Rouhi, 2020; Zhang et al., 2025).

### 5.2 Constructing an AIS spine FE model integrated with BMD information

The construction of a FE model for the AIS spine that integrates BMD data is a multi-step process that begins with the acquisition and processing of medical images. The foundational step involves segmenting spinal vertebrae from CT scans to reconstruct a patient-specific three-dimensional geometric model (Han et al., 2023). This three-dimensional reconstruction is crucial for capturing the complex, deformed anatomy of the scoliotic spine, including lateral curvature and axial rotation (Grünwald et al., 2021). Following geometry creation, the model is discretized into a mesh of FE, typically tetrahedral or hexahedral, to enable numerical analysis (Han et al., 2023). A critical and sophisticated phase is the assignment of material properties. To create a biomechanically realistic model, especially one that reflects osteopenia or osteoporosis often associated with AIS, the grayscale HU values from QCT scans are converted into spatially varying elastic moduli and strength properties for the bone tissue (Han et al., 2023). This conversion, based on established density-modulus relationships, allows the model to incorporate the heterogeneous bone quality distribution, such as the higher BMD often found on the concave side of the curve apex (Han et al., 2023). For models utilizing DXA, the areal BMD can be used to derive homogenized material properties, though with less spatial detail than QCT. Advanced implementations, such as those used in Biomechanical CT, perform FEA directly on clinical-resolution CT scans to measure bone strength and provide DXA-equivalent T-scores, offering a powerful opportunistic method for assessing fracture risk (Keaveny et al., 2020). The model’s completeness requires the incorporation of soft tissues. Ligaments are often modeled as nonlinear spring elements, while simulating muscle forces presents a greater challenge. Hybrid modeling approaches combine musculoskeletal models, which estimate muscle and reaction loads through optimization algorithms, with detailed FE models of the spine to investigate the effects of muscle activity or paralysis on stress distributions in growth plates or vertebrae (Kamal and Rouhi, 2020). Finally, physiological loading and boundary conditions are applied. This typically involves applying a compressive follower load along the spinal column to simulate body weight and moments in flexion, extension, lateral bending, and axial rotation to assess the spine’s range of motion and internal stress states under various activities (Zhang et al., 2025). For specific applications like brace simulation, external forces representing brace pads are applied to the torso model to predict corrective effects (Guy et al., 2024).

Integrating BMD data, particularly the volumetric and spatially resolved information from QCT, is paramount for creating a realistic “osteoporotic spine model” within the AIS context. This integration moves beyond a simple geometric representation to a model where the material degradation associated with low bone mass directly influences mechanical behavior. Studies have shown that the overall BMD in AIS patients can be lower than normal and exhibits a specific pattern, often decreasing from the cranial to caudal regions and showing asymmetry across the scoliotic curve (Han et al., 2023). By mapping these BMD variations to local elastic properties, the FE model can simulate the consequences of reduced bone quality. For instance, it can predict how decreased vertebral strength alters load-sharing patterns, potentially increasing stress concentrations on the concave side facet joints, which has been linked to subchondral sclerosis and hypertrophy in AIS (Yahara et al., 2023). Furthermore, integrating BMD allows for the investigation of mechanobiological interactions. Models can explore Frost’s Mechanostat theory in the AIS context, examining how the altered mechanical strain environment due to both deformity and BMD changes might influence bone remodeling responses (Hosseinzadeh-Posti et al., 2026). This is critical for understanding the vicious cycle of progression, where deformity leads to asymmetric loading, which may exacerbate bone loss in certain regions, further compromising stability. The model can also assess fracture risk more accurately than BMD alone; for example, CT-based FEA has been validated to predict incident vertebral fractures independently of the fracture’s location in the spine (Johannesdottir et al., 2021). In surgical planning, an integrated model can help evaluate the stability of different fixation constructs in osteoporotic bone, predict stress on pedicle screws, or optimize screw trajectories based on local BMD to enhance purchase and reduce the risk of pull-out (Han et al., 2023). Similarly, for brace treatment, understanding how corrective forces interact with vertebrae of varying density is essential for designing effective and safe orthoses that apply appropriate pressure without causing adverse effects on already compromised bone (Zhang et al., 2025).

Model validation is an indispensable step to ensure the reliability and clinical relevance of the simulation results. Validation typically involves comparing the outputs of the FE model against experimental data or established clinical observations. A common approach is to compare the model-predicted ROM or stiffness with data from *in vitro* biomechanical tests on cadaveric spine specimens under similar loading conditions (Joukar et al., 2022). For patient-specific models, validation can be performed by comparing simulated outcomes with *in vivo* measurements. For instance, the predicted immediate in-brace correction from a brace simulation model can be compared to the actual radiographic correction measured in the patient (Guy et al., 2024). One study evaluating a predictive brace action model reported that it successfully simulated 70% or more of patients within a preselected tolerance level for key geometrical parameters (Vergari et al., 2021). Another robust validation method involves comparing FE-predicted vertebral failure loads with experimentally measured failure forces from cadaveric testing, demonstrating strong correlations (e.g., r = 0.97) (Fereydoonpour et al., 2025). Furthermore, model validation can extend to verifying that the simulated internal mechanics align with known pathophysiology. For example, an AIS FE model that shows progressively increasing contact forces on the concave side facet joints from the mid to lower thoracic spine, peaking at the apex, validates against histological findings of facet joint sclerosis and hypertrophy in surgical specimens from AIS patients (Yahara et al., 2023). Validation also encompasses ensuring the material property assignment is accurate. Techniques like comparing synthetic areal BMD derived from QCT-based FE models with actual DXA measurements, or verifying that strain distributions match expectations, are employed (Xu J. et al., 2026). The emergence of artificial intelligence and automated pipelines further aids validation by ensuring anatomical accuracy through metrics like Dice coefficients (e.g., 93.7%) when comparing automated segmentations to manual gold standards, and by checking that simulated ROMs for both healthy and pathological FSU fall within ranges reported in the literature (Caprara et al., 2021). Ultimately, a rigorously validated FE model integrated with BMD data becomes a powerful digital twin, enabling clinicians to explore “what-if” scenarios, optimize treatment plans, and gain deeper insights into the biomechanical pathogenesis of AIS progression in the setting of compromised bone health (Alassaf et al., 2024).

## 6 Study on the influence of osteoporosis on the mechanical properties of AIS spine based on FEA

### 6.1 Impact of osteoporosis on the mechanical strength of vertebral bodies and spinal segments

FEA based on CT imaging provides a powerful tool to quantify the detrimental impact of osteoporosis on vertebral bone strength. Simulation studies consistently demonstrate that under identical mechanical loading conditions, osteoporotic vertebral bodies exhibit significantly elevated maximum stress concentrations and a markedly reduced failure load—the load at which compressive fracture initiates (Wu et al., 2021). This inverse relationship between increased stress and decreased load-bearing capacity directly indicates a compromised structural integrity and heightened fracture risk. The correlation between traditional bone mass indices, such as BMD, and FEA-predicted vertebral strength is often only weakly positive, suggesting that BMD alone is insufficient to accurately predict fracture risk (Wu et al., 2021). Biomechanical CT analysis, which predicts bone strength via CT-based FEA, has been validated as superior to DXA-measured areal BMD in predicting experimentally measured vertebral stiffness and strength, offering a more reliable assessment of mechanical competence (Wei et al., 2021). This is particularly relevant in clinical scenarios, as automated FEA modeling can reveal gender-specific differences, with women over 50 years showing higher vertebral strain and stress under the same applied force compared to men, highlighting demographic variations in biomechanical vulnerability (Chen HH. et al., 2025).

At the level of the FSU, which includes two adjacent vertebrae and the intervening disc, osteoporosis induces a reduction in segmental stiffness. This biomechanical alteration manifests as increased displacement during fundamental spinal movements, including axial compression, flexion, extension, and lateral bending. The diminished stiffness reflects a loss of structural resistance to deformation, leading to segmental hypermobility. This excessive motion is not isolated but can accumulate across multiple spinal segments, contributing to macroscopic spinal instability. The risk is particularly pronounced in the context of AIS, where the biomechanical environment is already altered. On the concave side of the spinal curvature, vertebral bodies are subjected to sustained high compressive stresses. When this elevated mechanical demand coexists with the low bone strength characteristic of osteoporosis, a critical risk scenario emerges. The combination creates a predisposition for vertebral microfractures and progressive deformity (Rezaei et al., 2021). Research indicates that QCT-based FEA of a single vertebra can effectively predict fracture outcomes for multi-level spinal segments, explaining a substantial proportion (70%–84%) of the variability in experimentally measured fracture strength, thereby underscoring the link between local bone weakness and segmental failure (Rezaei et al., 2021). Furthermore, regional heterogeneity within a vertebra is significant; the superior trabecular structure of the lumbar vertebral body possesses greater biomechanical susceptibility, with lower bone volume, BMD, and compressive force limits compared to the inferior region, confirming its dominant role in the initiation of osteoporotic compression fractures (Shin et al., 2023). This segmental and regional vulnerability, characterized by reduced stiffness, increased displacement, and lower failure thresholds, underpins the pathway from osteoporotic bone quality to progressive spinal instability and deformity progression.

### 6.2 Simulation of the impact of osteoporosis on overall spinal stability and scoliosis progression risk in AIS

FE method provides a powerful computational tool to simulate the biomechanical consequences of osteoporosis on the AIS spine under various loading conditions (Naoum et al., 2021). Studies employing full-spine FE models to simulate daily activities such as standing, walking, and bending have demonstrated that under osteoporotic conditions, stress concentration phenomena at the apical and end vertebrae of the scoliotic curve become more pronounced (Zhang et al., 2023). Specifically, research analyzing osteoporotic AIS lumbar models has shown that suffering from osteoporosis significantly increases the stress within the lumbar spine, with the increase being more obvious in AIS models compared to non-scoliotic models, and the stress distribution becomes more concentrated (Zhang et al., 2023). This heightened and localized stress, particularly at the curve apex, is a critical biomechanical factor that can compromise spinal stability. The evaluation of BMD using patient-specific three-dimensional FE models reconstructed from CT scans has further quantified these regional variations, with findings indicating that the highest BMD is often measured on the concave side of the apical vertebra, though significant side-to-side differences may not always be present (Han et al., 2023). This detailed mapping of BMD and associated material property degradation in FE method allows for a more accurate simulation of how reduced bone strength exacerbates stress concentrations, thereby increasing the risk of structural failure and instability during physiological loads.

However, it is critical to note that the assignment of osteoporotic material properties in FEA studies is not uniform. Many studies adopt a simplified approach, globally reducing the Young’s modulus of cortical and cancellous bone to represent overall BMD loss, often using density-modulus relationships such as those derived from Keyak or Morgan. While straightforward, this method fails to capture the anisotropic nature of bone tissue and does not account for the deterioration of bone microarchitecture (e.g., trabecular thinning, connectivity loss) that accompanies osteoporosis. In contrast, more sophisticated studies utilize anisotropic constitutive models, which incorporate orientation-dependent mechanical behavior, or they explicitly model trabecular structure via micro-CT-based representations to simulate the effects of microarchitectural degradation. These advanced approaches can yield substantially different stress distributions and failure predictions compared to simple isotropic, globally reduced modulus simulations. For instance, an isotropic reduction may overestimate stress concentrations at the concave side relative to anisotropic models, which better distribute loads through preferential trabecular orientations. Therefore, any interpretation of FEA results regarding osteoporosis effects on AIS must critically evaluate the material property assignment method used; the choice between isotropic vs. anisotropic models and between density-modulus equations can profoundly influence the simulated mechanical environment and subsequent conclusions about curve progression risk.

Furthermore, these computational models can be utilized to predict how osteoporosis might influence scoliosis progression during growth by simulating the Hueter-Volkmann law. This law states that increased pressure on a growth plate inhibits growth, while reduced pressure stimulates it (Driscoll et al., 2009). In a scoliotic spine, the concave side of the vertebral body experiences higher compressive stresses. FE models that incorporate osteoporotic material properties and simulate growth plate mechanics can predict that the asymmetric stress distribution across vertebral growth plates becomes more severe with osteoporosis (Zhang et al., 2023). The increased and more concentrated stress on the concave side, as simulated in osteoporotic AIS models, would theoretically lead to greater inhibition of growth on that side compared to a spine with normal BMD (Zhang et al., 2023). Conversely, the relatively lower stress on the convex side could allow for relatively less inhibited or even stimulated growth. This exacerbated asymmetric growth driven by the worsened mechanical environment can be modeled to simulate a progressive increase in the Cobb angle over time. The FE method thus serves as a virtual laboratory to test the hypothesis that low BMD is not merely a comorbid finding in AIS but a potential driver of curve progression by amplifying the biomechanical forces that dictate vertebral growth patterns.

Nevertheless, while the theoretical framework linking the Hueter-Volkmann law to osteoporosis-driven progression is compelling, the computational evidence directly demonstrating this effect remains sparse and requires more rigorous synthesis. Specifically, a critical question is how lower vertebral stiffness under osteoporotic conditions alters endplate mechanical strain thresholds during the peak growth velocity period. Current studies often simulate static or quasi-static loads (e.g., standing, bending) and report global stress metrics such as peak von Mises stress, but they rarely provide quantitative data on local endplate strains or analyze strain distributions across the growth plate during dynamic loading representative of daily activity during growth. Moreover, few models incorporate time-dependent growth modulation algorithms that directly link local mechanical strain to growth rate changes, as opposed to simply inferring growth inhibition from stress asymmetries. To rigorously test the hypothesis, computational studies must: (1) implement validated growth plate constitutive laws that relate strain magnitude to growth reduction, (2) subject models to physiologically relevant loading protocols simulating peak growth velocity activities (e.g., high-impact movements in adolescents), and (3) report local endplate strain distributions, not just global stresses. Without such quantitative evidence, the claim that osteoporosis directly accelerates Cobb angle progression via the Hueter-Volkmann mechanism remains a plausible but unconfirmed theoretical construct.

Through parametric studies within the FE framework, it is possible to begin quantifying the potential biomechanical relationship between the degree of BMD reduction and the rate of Cobb angle increase, thereby constructing a theoretical model for risk assessment. By systematically varying the Young’s modulus of cortical and cancellous bone to represent a spectrum from normal BMD to osteopenia and osteoporosis, researchers can observe corresponding changes in spinal displacement, stability, and stress distribution (Zhang et al., 2023; Guo et al., 2025). For instance, studies have shown that under the same load, the displacement of the lumbar spine increases and stability decreases with osteoporosis, with this effect being more pronounced in AIS spines (Zhang et al., 2023). This quantifiable loss of stability under decreasing BMD provides a mechanical correlate for progression risk. While direct longitudinal simulation of Cobb angle progression over years is complex, parametric analyses can link specific reductions in BMD (or its surrogate, material stiffness) to measurable increases in apical vertebral stress, endplate stress, and segmental ROM—all indicators of a less stable mechanical state conducive to progression (Guo et al., 2025). The development of tools like the MRI-based S1 VBQ score, which shows a moderate correlation with QCT BMD measurements in AIS patients, offers a clinically accessible parameter that could be integrated into such models (Yang et al., 2025a). By establishing correlations between clinical BMD assessments (e.g., VBQ score or Z-score), FE-derived biomechanical metrics (e.g., peak von Mises stress at the apex), and observed clinical progression rates, a predictive theoretical model can be formulated. This model would aim to stratify patients based on their biomechanical risk profile, where a lower BMD translates to a higher predicted rate of stress-mediated asymmetric growth and Cobb angle deterioration, ultimately informing more personalized monitoring and intervention strategies.

## 7 Simulation study on the biomechanical effects of osteoporosis on non-surgical treatment (brace treatment) for AIS

### 7.1 Biomechanical principles of bracing and their implementation in FE models

Bracing functions as a primary conservative intervention for AIS by applying external corrective forces to the trunk, thereby altering the load distribution on the spine to slow or halt curve progression (Grivas et al., 2022). The fundamental biomechanical principle involves the strategic placement of pressure pads and relief zones to generate a three-point force system, which aims to correct lateral displacement and induce derotation of the vertebrae (Bakhshian Talkhoncheh and Beigzadeh, 2026). FEA provides a powerful computational framework to model this complex interaction by integrating a detailed brace model with a patient-specific representation of the torso, spine, ribcage, and soft tissues (Koutras et al., 2021). Within such a model, the brace-trunk interface, including contact mechanics and the precise application of forces, can be simulated to predict immediate in-brace correction (Vergari et al., 2021). Researchers can explicitly define the position, direction, and magnitude of these corrective push vectors on the model’s surface, allowing for a systematic investigation of how different brace design features—such as pad placement, strap orientation, and sagittal contouring—contribute to three-dimensional spinal realignment (Guy et al., 2022). For instance, studies have shown that pressure points applied at the level of the apical vertebra provide superior correction of the main thoracic curve compared to more caudal locations, and that ventral supports can inadvertently flatten lumbar lordosis (Guy et al., 2022). The implementation of bracing mechanics in FEA also enables the exploration of differential biomechanical effects. By varying material properties within the spinal model, such as the stiffness of intervertebral discs, vertebrae, and ligaments, simulations can reveal how spinal flexibility influences the corrective outcome, with intervertebral disc parameters having a greater effect on Cobb angle correction and bone parameters more significantly affecting sagittal alignment (Wei et al., 2023). This capability is crucial for hypothesizing how osteoporosis, characterized by reduced BMD and altered material properties, might affect force transmission and correction efficacy. Models can be personalized to represent osteoporotic bone by adjusting the elastic modulus of cancellous and cortical bone, allowing analysts to compare stress distributions, vertebral deformations, and achievable corrections against a model with normal BMD (Wei et al., 2022). Furthermore, advanced FEA workflows not only simulate the immediate mechanical action but also incorporate long-term growth modulation, modeling how sustained brace forces influence epiphyseal growth plates over treatment periods, which is essential for predicting final outcomes (Guy and Aubin, 2023). The integration of optimization algorithms with differentiable biomechanics represents a significant advancement, enabling the automated, simulation-driven design of brace geometry that seeks an optimal trade-off between clinical correction (e.g., maximizing Cobb angle reduction and derotation) and patient comfort (e.g., minimizing interface pressure) (Kardash et al., 2022). This approach has been validated in synthetic studies, showing that algorithmically designed braces can improve clinical metrics by up to 45% on average (Kardash et al., 2022). Moreover, the combination of CAD/CAM manufacturing with FEA simulation facilitates the creation of patient-specific digital twins, allowing clinicians to virtually test and refine brace designs—including novel flexible or three dimensional-printed braces—before physical fabrication, thereby optimizing the biomechanical prescription (Guy et al., 2024; Liu et al., 2024). This computational methodology provides a controlled environment to dissect the individual contributions of various passive correction mechanisms, offering objective insights that are difficult to obtain clinically and paving the way for more effective, personalized bracing strategies tailored to the patient’s unique spinal biomechanics and bone quality (Guy et al., 2022; Li D. et al., 2025).

### 7.2 Biomechanical analysis of the impact of osteoporosis on brace treatment efficacy

Simulation studies suggest that for the osteoporotic AIS spine, the same brace corrective force may lead to higher local stress in the vertebral body-brace contact area, increasing the risk of skin pressure ulcers and patient discomfort. The fundamental biomechanical principle underlying this risk is the altered material properties of osteoporotic bone, characterized by reduced BMD and compromised structural integrity. This results in a lower modulus of elasticity and yield strength, meaning the bone is less stiff and more prone to deformation under load. When an external orthotic force is applied through a brace pad, the force is distributed over a contact area. In osteoporotic vertebrae, the reduced load-bearing capacity means that the same magnitude of corrective force generates higher stress (force per unit area) at the bone-pad interface and within the underlying trabecular bone. This elevated stress concentration, which can be quantified through FEA modeling, not only risks microfractures in the vertebral endplates or bodies but also translates to higher pressure on the overlying soft tissues and skin. Prolonged exposure to such elevated pressures is a well-documented precursor to tissue ischemia and pressure ulcer formation, directly impacting patient compliance and tolerance.

It is important to clarify that these risks are specific to the context of AIS with concomitant low bone density, which involves a growing skeleton with pubertal bone accrual deficits. This pathophysiology is distinct from degenerative lumbar scoliosis or adult spinal deformities, where age-related bone loss and structural degeneration (e.g., disc collapse, facet arthropathy) are the primary drivers of bone fragility. While comparative analogies to adult populations can provide useful biomechanical insights, they must be carefully delineated to avoid confusion regarding age-specific bone pathophysiology. For example, in patients with degenerative lumbar scoliosis, significantly lower HU values, indicative of lower BMD, are commonly observed (Sun et al., 2025). This compromised bone quality in adults reflects a different biological process—senile or postmenopausal osteoporosis—rather than the deficits in peak bone mass acquisition seen in AIS. Similarly, research indicates that osteoporosis is a significant independent risk factor for complications in spinal instrumentation, including screw pull-out and adjacent segment fractures, highlighting the general fragility of the osteoporotic spinal column under mechanical loads (Mugge et al., 2022). However, in the AIS population, fragility is often relative, as adolescents typically have higher baseline BMD than elderly patients, and the concern is more about compromised force tolerance rather than outright structural collapse. This inherent fragility extends to the interface with external bracing, where the goal of applying corrective forces must be balanced against the risk of iatrogenic injury to both bone and soft tissue.

A more mechanistic understanding of force transmission in the osteoporotic spine reveals that reduced bone stiffness critically alters the balance between two competing biomechanical objectives: corrective trunk translation and the induction of localized, often irreversible, plastic deformation. In a healthy spine, the applied brace force predominantly results in elastic deformation of the spinal column, achieving translation of the vertebral bodies toward the midline while the bone and endplates remain within their elastic range. However, in osteoporosis, the lowered modulus of elasticity and ultimate strength mean that the same magnitude of corrective force, intended for trunk translation, can instead be partially dissipated through localized yielding of the vertebral endplates and the ribcage. Specifically, as the brace pad pushes against the rib hump, the force must be transmitted through the ribs to the costovertebral joints and then to the vertebral bodies. In osteoporotic bone, the ribs and endplates may undergo plastic deformation (e.g., microfracture of the trabecular network or buckling of the cortical shell) before sufficient force is transmitted to achieve the desired spinal derotation or translation. FE models can simulate this by incorporating elastic-plastic material laws for bone, differentiating between normal and osteoporotic tissue. Such models would show that the energy imparted by the brace is partitioned: a smaller proportion goes into elastic trunk translation and intervertebral disc compression, while a larger proportion is absorbed as inelastic damage (plastic strain) in the cancellous bone of the vertebral bodies and rib heads. This not only reduces the efficiency of correction—because the force applied does not translate fully into curve reduction—but also introduces a risk of persistent structural deformation (e.g., permanent vertebral body wedging or rib deformation) that could compromise long-term spinal mechanics and increase pain. Consequently, the mechanical “failure” of the osteoporotic brace interface is not merely about force loss through viscoelastic dissipation; it is about the onset of plastic damage that both undermines correction and creates iatrogenic morbidity.

Due to the poorer “baseline” stability of the osteoporotic spine, braces may struggle to effectively transmit corrective forces to the target vertebrae, potentially reducing orthotic efficiency. Models can analyze the transmission and dissipation of corrective forces within the spine. The biomechanical efficacy of a spinal brace relies on creating a three-point bending system to apply moments that correct the coronal and axial plane deformities. This requires a stable foundation; the corrective forces generated at the brace pads must be transmitted through the rib cage, soft tissues, and ultimately to the vertebral bodies to induce derotation and realignment. In osteoporosis, the reduced stiffness of the vertebral bodies and the entire spinal column increases its compliance. This increased flexibility might seem advantageous for correction, but it comes at the cost of structural stability. Under load, the osteoporotic spinal segments exhibit greater viscoelastic deformation and energy dissipation. Computational models, such as patient-specific FE models incorporating heterogeneous material properties based on BMD data from QCT or DXA, can visualize this phenomenon. They would show that a significant portion of the applied brace force is absorbed in deforming the compliant vertebral bodies and intervertebral discs rather than being efficiently transmitted along the spinal column to generate the intended corrective moment at the apex of the curve. This energy dissipation manifests as a loss of corrective force fidelity, meaning the force measured or intended at the brace pad does not equate to the effective force acting on the target vertebral geometry.

Again, these phenomena must be contextualized for AIS. In adolescent patients, the spine is still growing, and the intervertebral discs are more hydrated and resilient compared to the degenerated discs typical of degenerative lumbar scoliosis. Thus, while osteoporotic AIS bone may be more compliant, the overall spinal column in adolescents retains greater viscoelastic recovery and lacks the significant disc degeneration that further limits force transmission in adults. The concept of poor force transmission is indirectly supported by clinical observations in related fields, but only when the relevant age-specific differences are noted. For example, in the context of vertebral rotatory subluxation in degenerative lumbar scoliosis, a condition reflecting severe spinal instability, there is a strong association with both osteoporosis and asymmetric paraspinal muscle degeneration (Wang M. et al., 2025). This combination of poor bone quality and impaired dynamic muscular stabilization represents an extreme example of compromised “baseline” stability, making any external corrective intervention challenging. However, in AIS, paraspinal muscle function is generally intact, and stability deficits are primarily due to bone quality rather than soft tissue degeneration. Similarly, studies on surgical outcomes note that osteoporosis is a risk factor for instrumentation failure, indicating that internal fixation struggles to maintain purchase in poor-quality bone (Mugge et al., 2022). While this analogy is useful for understanding mechanical principles, it is important to note that in AIS, the bone is often less severely osteoporotic than in elderly patients, and screw pull-out risks are lower albeit still elevated compared to normal bone. By analogy, external bracing faces a similar challenge: achieving and maintaining a stable mechanical purchase on a deformable structure to transmit forces effectively. Biomechanical models can specifically analyze parameters like intervertebral force vectors, stress distribution across disc spaces, and the resultant changes in Cobb angle per unit of applied brace force, comparing these metrics between models with normal and osteoporotic bone properties to quantify the efficiency loss.

These analyses aid in optimizing brace design, for instance, by adjusting the position and pressure distribution of force pads for patients with low BMD to achieve personalized brace therapy. The insights gained from biomechanical modeling directly inform clinical adaptations. Recognizing that osteoporotic vertebrae cannot withstand high, localized pressures, brace design can be modified to distribute forces over a larger surface area. This could involve using larger, contoured pads or dynamic pressure-relieving materials that adapt to the patient’s anatomy. More critically, FEA can guide the strategic placement of pads. Instead of applying maximum force directly over the most prominent vertebral bodies (which may be the most osteoporotic), models might indicate that applying controlled forces at specific levels above and below the apex, or using a combination of derotational and translational pads, achieves the desired correction with lower peak stresses on any single vertebra. This approach aligns with the principle of load sharing to protect vulnerable bone. Personalization is key, as BMD can vary significantly along the spine, especially in scoliosis where asymmetric loading may lead to differential osteoporosis. Research in degenerative lumbar scoliosis patients shows that CT HU values, a marker of BMD, tend to be lowest at the apex of the scoliotic curve, and this pattern is more pronounced in severe curves (Xu F. et al., 2026). While this finding is from an adult population, it suggests a pattern that may also apply to AIS, where asymmetric mechanical loading during growth could lead to localized BMD deficits. A personalized brace for an AIS patient with low BMD would ideally integrate such spatially specific bone quality data, perhaps derived from low-dose EOS imaging or other modalities, to map regions of high and low resistance. Force application could then be concentrated on vertebral segments with relatively better bone stock. Furthermore, the optimization extends to treatment protocols. For osteoporotic patients, a slower, more gradual correction regimen with frequent monitoring might be preferable to allow bone remodeling to adapt to the new loads, reducing the risk of pain and compression. However, in growing AIS patients, there is a window of opportunity for bracing that is limited by skeletal maturity; thus, the trade-off between gradual correction and timely intervention must be carefully considered. The ultimate goal is to develop a “BMD-aware” bracing strategy that maximizes corrective biomechanical efficiency while minimizing the risks of skin breakdown, patient discomfort, and potential vertebral injury, thereby improving the therapeutic window and outcomes for AIS patients with concomitant low bone density.

## 8 The impact of osteoporosis on surgical treatment strategies and internal fixation stability for AIS

### 8.1 Preoperative BMD assessment and surgical decision-making

Severe osteoporosis is considered a relative contraindication for spinal deformity correction surgery due to its significant association with an increased risk of internal fixation failure, such as screw cut-out, pull-out, and rod breakage, as well as postoperative loss of correction. This risk is underpinned by the well-documented prevalence of low BMD in patients with AIS. Multiple studies have established a clear correlation between lower BMD and greater curve severity. For instance, a study involving 348 surgical AIS patients found that those with low BMD (Z-score ≤ −1) had significantly larger preoperative Cobb angles (59.3° vs. 51.4°) and lower BMI compared to those with normal BMD (Shibata et al., 2025). This inverse relationship is further supported by longitudinal data showing that AIS patients with low preoperative BMD are likely to have persistent low BMD at skeletal maturity, and this persistent low BMD is associated with more severe preoperative curves (Nishida et al., 2023). The association is not limited to DXA measurements; studies using QCT have also demonstrated a significant negative correlation between BMD and both Cobb angle and vertebral rotation angle in AIS patients (Li et al., 2024). Furthermore, research indicates that the asymmetrical loss of vertebral BMD, assessed via HU, aggravates with curve progression, showing significantly lower density on the convex side of the curve apex (Cheng et al., 2022). This systemic and often persistent osteopenia in AIS (Li et al., 2020) directly compromises the biomechanical purchase of spinal implants, making preoperative BMD assessment a critical component of surgical planning to mitigate the risk of mechanical complications.

FE modeling serves as a powerful tool for preoperative planning by simulating the biomechanical effects of various surgical parameters under different BMD conditions. These computational models can evaluate the performance of different screw designs—such as variations in diameter, length, and thread pattern—and compare different implantation trajectories, including traditional pedicle screws and cortical bone trajectory screws, under applied corrective forces. The utility of such models is enhanced by advanced methods for assessing bone quality. For example, three-dimensional FE modeling based on CT reconstruction has been used to evaluate site-specific BMD in AIS vertebrae, providing detailed insights that are valuable for surgical planning and biomechanical analysis (Han et al., 2023). Additionally, opportunistic screening tools like the MRI-based VBQ score, including adaptations for the sacral (S1) or cervical vertebrae, offer a novel means to assess BMD without additional radiation, showing significant correlation with QCT-measured BMD and potential for identifying low BMD preoperatively (Yang et al., 2025a; Yang et al., 2025b). These assessment methods feed critical data into FE models, allowing surgeons to virtually test how different fixation constructs will behave in osteoporotic bone, thereby informing the selection of optimal screw type and trajectory to maximize pull-out strength and stability.

Through comparative simulation, FEA can guide the selection of superior screw types, enhanced fixation techniques, or the decision for a staged surgical strategy for AIS patients with osteoporosis. The simulations can identify areas of high stress concentration and potential failure, prompting the consideration of augmentation techniques like cement-augmented screws to improve fixation strength in low-density bone. The clinical decision-making process is informed by the understanding that low BMD is a significant risk factor. A large retrospective study of 798 surgical AIS patients found a high prevalence of low BMD (Z-score ≤ −1 in 37.5% of patients), which was more severe and prevalent in boys than in girls with severe curves, suggesting BMD may be a more valuable predictive factor for progression in males (Wu et al., 2023). Therefore, for a patient with severe scoliosis and concomitant osteoporosis, FE simulations might demonstrate that standard pedicle screws would yield unacceptably high strain under corrective forces. This could lead to the preoperative decision to use cement-augmented screws, particularly on the convex side around the apex where asymmetrical bone loss is greatest (Cheng et al., 2022), or to select a larger-diameter screw design. In extreme cases, if simulations indicate a high probability of catastrophic failure with a single-stage correction, the model may support planning a staged procedure with initial anterior release and fusion to reduce mechanical demand on the posterior instrumentation. Ultimately, integrating detailed preoperative BMD assessment—whether by DXA, QCT, HU, or MRI-VBQ—with patient-specific FE modeling enables a personalized, biomechanically rational approach to surgical planning, aiming to optimize construct stability and long-term outcomes in the challenging cohort of osteoporotic AIS patients.

### 8.2 Application of FEA in assessing postoperative stability

FEA has become an indispensable tool for evaluating the biomechanical stability of the spine following internal fixation surgery, particularly in the context of osteoporosis. By constructing integrated FE models of the postoperative spine-instrumentation system, researchers can perform detailed analyses of stress distribution at the critical screw-bone interface. This is vital for predicting regions at high risk for screw loosening and pullout in osteoporotic vertebrae. For instance, studies have shown that in osteoporotic or osteolytic conditions, the stress on pedicle screws, especially under axial pullout forces, is significantly elevated (Nevzati et al., 2024). Comparative FEA of different anti-osteoporosis medications, such as denosumab and bazedoxifene, has demonstrated that denosumab more effectively enhances pedicle screw fixation strength, particularly against axial and cranial/lateral-directed loads, by improving overall vertebral compression strength (Asada et al., 2025). Furthermore, the analysis of different pedicle screw designs and insertion techniques in degenerated bone reveals that factors like screw diameter and trajectory significantly influence stress at the bone-screw junction, with larger diameter screws (e.g., 6.0 mm) generally exhibiting lower stress concentrations, which is crucial for stability in low-density bone (Jain and Khan, 2021). These models allow for the precise simulation of the spine’s immediate post-fusion stability, enabling the assessment of how different surgical correction strategies affect the residual spinal segments. A critical concern is the accelerated degeneration of adjacent segments, a risk that is magnified in osteoporotic spines due to altered load transfer. FEA studies comparing various reconstruction techniques for conditions like thoracolumbar burst fractures or osteolytic lesions have quantified the mechanical burden on the proximal junctional level. For example, while long-segment posterior instrumentation provides greater global motion reduction, it can increase intradiscal pressure and stress at the proximal adjacent level, potentially hastening proximal junctional kyphosis (PJK) (Wong et al., 2022). Conversely, three-column spinal reconstruction techniques, which combine anterior support (e.g., with a cage or bone cement) with posterior instrumentation, often provide better stabilization at the index level while offloading the proximal junction, thereby reducing the risk of PJK (Wong et al., 2022). The analysis of novel rod designs, such as Bezier surface-smoothed transition rods, further supports this, showing that optimized load distribution can significantly reduce stress on the upper adjacent vertebrae and pedicle screws compared to constant-diameter rods (Le Naveaux et al., 2025). These computational insights are foundational for engineering improvements in implant design and surgical technique to bolster long-term postoperative stability in osteoporotic spines. Research into alternative fixation methods, such as a novel cortico-pedicular posterior fixation device or a self-locking oblique lateral interbody fusion system, demonstrates through FEA that these constructs can provide stability comparable to traditional pedicle screw systems while potentially reducing stress on the instrumentation and adjacent segments (Eastlack et al., 2023; Chen et al., 2026). Similarly, the biomechanical evaluation of different interbody cage types, including trussed titanium cages designed for direct osseointegration, highlights how implant design influences the early segmental stability and bone ingrowth process, which is critical for achieving solid fusion in compromised bone (Loenen et al., 2022). The application of FEA extends to optimizing surgical parameters, such as determining the effective screw-to-cage length ratio for stand-alone lateral lumbar interbody fusion devices to ensure adequate torsional stability without supplemental posterior fixation (Malham et al., 2025). Ultimately, these sophisticated analytical models bridge the gap between mechanical engineering principles and clinical practice, offering a powerful platform to preoperatively plan and personalize surgical strategies, select appropriate implants, and mitigate complications associated with internal fixation in the challenging biomechanical environment of osteoporotic bone.

## 9 Challenges and future directions in integrated research

### 9.1 Multiscale modeling and multiphysics coupling

The primary challenge in understanding the interplay between osteoporosis and curve progression in AIS lies in effectively coupling macroscopic spinal biomechanics with microscopic bone remodeling biology. Current modeling approaches often treat these scales in isolation, limiting the dynamic simulation of their interaction. A promising direction involves integrating organ-level FE models of the scoliotic spine with tissue- and cell-level biological models of bone adaptation. For instance, one study developed a unified computational framework that couples a cell population dynamics model, based on Komarova’s formulation, with a mechanical stimulus model derived from strain energy density calculations, using a discrete one dimensional FE approach to represent trabecular bone structure (Quexada et al., 2023). This model successfully links mechanical loading to the spatio-temporal dynamics of osteoclasts and osteoblasts, allowing the simulation of BMD evolution in pathological states like osteoporosis. However, scaling such a detailed cellular model to the entire organ level of a deforming spine remains computationally intensive and conceptually complex. The challenge is to create a feedback loop where the altered mechanical environment of a scoliotic spine—characterized by asymmetric loading—directly influences bone cell activity, leading to localized bone loss (osteoporosis) or formation, which in turn further modifies the spinal structure’s mechanical stability and propensity for curve progression. This requires a multiscale FE framework that can seamlessly transfer information on mechanical stimuli (e.g., strain, fluid flow) from the macro-scale (vertebral bodies, discs) down to the micro-scale of bone trabeculae and the cellular microenvironment.

Future advancements necessitate the development of sophisticated multiscale, multiphysics FE models that span from the organ (entire spine) to the tissue (cancellous and cortical bone) and down to the cellular level. A critical component is the incorporation of fluid-structure interactions, particularly within the intervertebral disc, as its mechanical behavior and nutrient transport significantly influence adjacent vertebral bone health. The integration of different physical fields—solid mechanics, fluid dynamics, and biochemical signaling—is essential. Research has demonstrated the value of spatio-temporal multiscale approaches combining longitudinal *in vivo* micro-CT with subject-specific FE modeling to map bone adaptation at high resolution in response to combined mechanical and pharmacological interventions (Cheong et al., 2021). This work highlighted region-dependent interactions between therapies, underscoring the need for models that can resolve such local effects within a complex spinal geometry. Furthermore, agent-based modeling coupled with micro-multiphysics simulations, as used to investigate post-denosumab bone loss, represents a powerful tool for this integration (Ledoux et al., 2025). Such models can simulate the behavior of individual bone cells (agents) in response to a mechanically and biochemically defined microenvironment, revealing key drivers of bone turnover. For AIS, a future model must also account for growth. The ultimate goal is a patient-specific digital twin that can simulate the progression of scoliosis in conjunction with BMD changes, incorporating factors like hormonal influences, genetic predisposition, and mechanical loads from daily activities. This would enable the prediction of high-risk scenarios for curve progression and osteoporotic fracture, allowing for personalized, preemptive interventions. The technical pathway involves leveraging advanced imaging (e.g., MRI, HR-pQCT) to inform geometry, employing concurrent or hierarchical multiscale computational schemes, and validating model predictions against longitudinal clinical data on both curve magnitude and BMD in AIS cohorts.

### 9.2 Personalized and precision medicine applications

The future trajectory of AIS management is decisively oriented towards personalized and precision medicine, leveraging advanced computational and data integration technologies to enhance predictive accuracy and therapeutic decision-making. A pivotal trend involves harnessing artificial intelligence (AI) to accelerate the generation of personalized FE models, which can be integrated with multi-omics data—including genomics and proteomics—to construct sophisticated “digital twin” models of the spine. These digital replicas are envisioned to predict an individual patient’s risk of curve progression and optimize treatment pathways. This approach is grounded in the growing recognition that AIS is a multifactorial condition with a suspected polygenic basis, where systemic factors like bone metabolism play a crucial role (Shibata et al., 2025). For instance, genome-wide profiling has identified specific circulating microRNAs (e.g., miR-1-3p, miR-19a-3p, miR-19b-3p) that are upregulated in patients who develop severe scoliosis, enabling the development of algorithms with high accuracy, sensitivity, and specificity for predicting progression risk (Khatami et al., 2025). The integration of such biomarker data with biomechanical models derived from imaging could create a powerful predictive tool. Furthermore, research into rare genetic variants, such as those in the FNDC1 gene associated with severe AIS and altered BMD, underscores the potential for genomics to inform personalized risk stratification and elucidate pathophysiological mechanisms (Charng et al., 2025). The concept of a digital twin extends beyond genetics to incorporate detailed phenotypic data. For example, three-dimensional FE modeling based on CT scans has been used to evaluate vertebral-specific BMD in AIS patients, revealing patterns such as higher density on the concave side of the apex and an overall decrease in BMD from the cranial to caudal spine (Han et al., 2023). Integrating these spatially resolved biomechanical properties with omics data in an AI-driven framework could simulate how a specific patient’s spine, with its unique genetic predisposition and bone quality, will respond to mechanical loads or growth, thereby enabling pre-emptive and tailored interventions.

Parallel to the development of digital twins, there is a concerted effort to develop clinical decision support systems (CDSS) rooted in FEA and AI-driven predictive modeling. These systems aim to translate complex simulation predictions—such as the expected outcomes and associated risks of different bracing protocols, physiotherapeutic exercises, or surgical plans—into intuitive visualizations for clinicians, thereby facilitating the formulation of precise, individualized treatment strategies. The move towards precision medicine in AIS surgery, for example, seeks to inform patients of the likelihood of achieving specific quality-of-life outcomes, as measured by instruments like the Scoliosis Research Society-22r (SRS-22r) questionnaire (Stone et al., 2025). However, current predictive capabilities for individual SRS-22r item responses are limited, partly due to ceiling effects in the questionnaire, highlighting the need for more sophisticated, multi-parameter models (Stone et al., 2025). AI-enabled systems are being developed to address this gap. One study trained machine learning models on 171 pre-operative clinical features from 455 pediatric patients to predict post-operative SRS-22r scores at 6-month, 1-year, and 2-year intervals, achieving area under the curve performances of up to 0.86 (Shi W. et al., 2025). Explainability analysis within such systems can quantify the contribution of various pre-operative parameters, suggesting, for instance, that greater attention to sagittal alignment and patient self-image, beyond the major Cobb angle, could improve surgical decision-making (Shi W. et al., 2025). This responsible AI approach includes calibrating model confidence for human intervention and mitigating health disparities to ensure algorithmic fairness. For conservative management, predictive factors for successful non-operative treatment and achieving a minimal clinically important difference in health-related quality of life have been identified, including specific radiological (e.g., lateral flexion range <25°) and clinical parameters (e.g., higher anxiety/depression scores) (Cheung et al., 2022). A CDSS could integrate these predictors with FE simulations of brace mechanics or exercise regimens to recommend the most effective conservative strategy for a given patient profile. Moreover, mobile health technology demonstrates the feasibility of real-time monitoring of brace adherence and treatment-related experiences, with apps triggering clinical alerts for issues like emotional distress or pain, thereby enabling timely clinical responses and supporting personalized engagement strategies (Sánchez-Raya et al., 2025). The ultimate goal is a holistic CDSS that synthesizes data from genetic risk scores (Khatami et al., 2025), longitudinal BMD assessments (Nishida et al., 2023), biomechanical simulations (Han et al., 2023), patient-reported outcomes (Negrini et al., 2023), and real-time adherence metrics (Sánchez-Raya et al., 2025) to present clinicians with a comprehensive, evidence-based projection of treatment efficacy, empowering shared decision-making and optimizing long-term patient outcomes in the era of personalized orthopedic care.

## 10 Clinical translation and comprehensive management strategies

### 10.1 Comprehensive intervention based on biomechanical understanding

Clinical management of AIS must evolve beyond the singular goal of morphological correction to incorporate the fundamental improvement of bone health as a core component of a holistic treatment strategy. This paradigm shift is critically supported by biomechanical evidence demonstrating the intrinsic link between compromised bone quality and spinal instability in AIS. Advanced imaging and modeling studies consistently reveal that AIS is frequently accompanied by systemic and localized osteopenia or osteoporosis. For instance, a study utilizing three-dimensional FE modeling based on CT reconstructions in AIS patients reported a mean global spine BMD of 0.83 ± 0.15 g/cm^2^, with a notable negative correlation between apical vertebral BMD and patient age and height (Han et al., 2023). This systemic reduction in BMD undermines the structural integrity of the vertebral column, making it more susceptible to deforming forces. The biomechanical vulnerability is further highlighted during the critical peri-pubertal peak height velocity (PHV) period. A comprehensive cross-sectional and longitudinal study of 396 girls with AIS found that at PHV, subjects exhibited significantly decreased cortical volumetric BMD, cortical thickness, and apparent modulus (a measure of bone mechanical strength) compared to both pre-PHV and post-PHV stages, alongside elevated bone turnover markers (Yang et al., 2022). This coupling of rapid growth with diminished bone strength provides a direct biomechanical link to the observed curve progression during this period, positioning low BMD as a key prognostic factor. Therefore, for AIS patients diagnosed with osteopenia or osteoporosis, proactive anti-osteoporotic intervention is not merely adjunctive but essential. This necessitates routine assessment of bone health using reliable tools. Opportunistic screening methods, such as the MRI-based S1 VBQ score, have shown promise, with an S1 VBQ score threshold of 3.2 demonstrating high sensitivity and specificity for identifying low BMD in AIS patients, offering a valuable pre-surgical screening tool without additional radiation exposure (Yang et al., 2025a). The intervention strategy should be multifaceted, beginning with ensuring adequate nutritional support, specifically sufficient calcium and vitamin D intake, to provide the foundational substrates for bone mineralization. Concurrently, prescribing safe, controlled weight-bearing physical exercise is paramount. Activities such as swimming and specific postural muscle-strengthening exercises serve a dual purpose: they apply beneficial mechanical loads to the skeleton to stimulate bone formation and improve BMD, while simultaneously enhancing the strength and endurance of the paraspinal and core musculature. Stronger muscles provide dynamic stabilization to the spine, offloading mechanical stress from the vertebrae and potentially mitigating asymmetric loading that contributes to curve progression. This integrated approach aligns with Frost’s Mechanostat theory, where mechanical strain regulates bone adaptation. Computational modeling investigations support this, showing how muscle forces influence vertebral BMD distribution in AIS (Hosseinzadeh-Posti et al., 2026). By fortifying both the passive (bone) and active (muscle) components of the spinal stabilizing system, such a comprehensive intervention aims to break the potential “vicious circle” where postural disorder increases spinal loads, which in turn exacerbates the disorder, as suggested by advanced biomechanical analyses like barycentremetry (Skalli et al., 2026). Ultimately, embedding bone health optimization into the standard care protocol for AIS, particularly for those with identified deficits, represents a proactive, biomechanically-informed strategy to enhance spinal stability, support other treatment modalities, and improve long-term musculoskeletal outcomes.

### 10.2 Building a multidisciplinary collaborative diagnosis and treatment model

The effective management of AIS complicated by osteoporosis necessitates a tightly integrated, multidisciplinary approach involving orthopedics, endocrinology, rehabilitation, nutrition, and bioengineering experts (Sheth et al., 2025). This collaborative framework is essential for addressing the complex interplay between spinal deformity and compromised bone health, which significantly elevates the risk of surgical complications, implant failure, and poor long-term outcomes (Bauer et al., 2025). The core of this model is a structured, patient-centered pathway that begins with comprehensive preoperative assessment and optimization. For instance, a case report of an 18-year-old male with extreme AIS (Cobb angle >180°) and severe malnutrition (BMI 14.7) demonstrated the critical role of a multidisciplinary team (MDT). The team implemented an 8-week presurgical optimization protocol involving halo-gravity traction, therapeutic exercises, respiratory rehabilitation, and nutritional intervention, all guided by patient-specific three dimensional-printed spinal modeling. This intervention led to quantifiable improvements in height, weight, pulmonary function, and pain levels, enabling a successful posterior spinal fusion (Chu, 2025). This case underscores that managing complex AIS, especially with comorbidities like osteoporosis, requires coordinated expertise beyond a single surgical specialty to mitigate risks and prepare the patient physiologically for major intervention.

The diagnostic and therapeutic workflow within this collaborative model must systematically integrate conventional BMD screening, imaging-based biomechanical risk assessment, and the formulation of a stepped, personalized treatment plan (Bauer et al., 2025). Routine BMD evaluation using modalities like QCT is paramount, as studies consistently show AIS patients have a higher prevalence of low BMD and osteopenia compared to healthy controls, which is correlated with greater curve severity (Shibata et al., 2025; Yang et al., 2023). Opportunistic screening using existing imaging, such as calculating VBQ scores from T1-weighted MRI of the first sacral vertebra (S1), has also been validated as a novel tool for assessing BMD in AIS patients, providing a practical method for preoperative risk stratification (Yang et al., 2025a). Future risk assessment may incorporate simplified indicators derived from FEA, a computational tool that can model the biomechanical effects of osteoporosis on the scoliotic spine, predicting areas of high stress concentration and increased fracture risk (Zhang et al., 2023). The treatment strategy should be tiered and individualized, encompassing foundational interventions like nutritional optimization (e.g., calcium and vitamin D supplementation), targeted physiotherapeutic scoliosis-specific exercises to improve muscle strength and potentially slow progression, and appropriate bracing (Catanzariti et al., 2024). For surgical candidates, meticulous planning is required, potentially involving techniques to enhance implant fixation in osteoporotic bone, such as cement augmentation or the use of hydroxyapatite granules to reinforce pedicle screws (Kanno et al., 2022; Schömig et al., 2023).

Regular monitoring of both BMD and scoliosis curve progression is crucial for dynamically adjusting the management strategy. Longitudinal studies indicate that low BMD can persist into bone maturity in a significant proportion of AIS patients and is associated with larger preoperative Cobb angles (Nishida et al., 2023). Therefore, ongoing surveillance is needed to identify patients at risk for accelerated bone loss or curve progression. The implementation of structured MDT meetings has been shown to significantly improve patient outcomes. A quality improvement study demonstrated that instituting monthly multidisciplinary conferences focusing on preoperative planning, level selection, and postoperative protocol optimization led to substantial benefits. These included reduced estimated blood loss, shorter operative times, fewer fused levels, earlier postoperative ambulation, lower complication rates, and decreased hospital length of stay for patients undergoing posterior spinal fusion for AIS (Hengartner et al., 2024). Furthermore, integrating such MDT collaboration into Enhanced Recovery After Surgery (ERAS) protocols has proven highly effective. Studies comparing ERAS pathways designed by MDT to traditional perioperative care show that the ERAS approach results in less blood loss, lower pain intensity, faster ambulation, and shorter hospital stays, facilitating rapid rehabilitation (Ding et al., 2022; Yang et al., 2020). The multidisciplinary model extends into the postoperative and long-term phases, including transitional care into adulthood. This involves surveillance for late complications (e.g., delayed hemothorax from vascular irritation), monitoring for adjacent segment degeneration—a risk heightened by osteoporosis—and addressing psychosocial wellbeing through targeted interventions (Alexeev et al., 2026; Wang L. et al., 2025; van Niekerk et al., 2023). For female patients of childbearing age, preconception counseling involving obstetricians and anesthesiologists is vital to plan for safe pregnancy and delivery options despite previous spinal instrumentation (Landrum et al., 2023). Ultimately, this comprehensive, adaptive, and team-based approach is key to optimizing skeletal health, spinal stability, and overall quality of life for AIS patients with concurrent osteoporosis.

## 11 Conclusion

The intricate relationship between AIS and osteoporosis represents a compelling nexus of clinical observation and pathophysiological inquiry. This review has synthesized evidence demonstrating that low BMD is a prevalent feature in AIS cohorts, suggesting a potential role in compromising spinal biomechanical integrity and thereby contributing to the initiation and progression of the spinal deformity. This conclusion synthesizes the key themes from the outlined research paradigm, analyzing the current landscape from an expert perspective, balancing diverse research findings, and charting a course for future investigation and clinical integration.

From a diagnostic and monitoring standpoint, the central role of BMD assessment technologies, particularly DXA and QCT, is unequivocal. These tools have transitioned from research instruments to essential clinical aids, providing the quantitative bedrock for identifying osteopenia/osteoporosis in AIS patients. Their findings are no longer mere ancillary data but are integral to risk stratification. However, an expert perspective necessitates a balanced view of their limitations. DXA, while widely available, is susceptible to projection errors in a deformed spine, potentially underestimating or overestimating true volumetric density. QCT offers superior volumetric assessment but involves higher radiation exposure and cost. The clinical challenge lies in judiciously selecting and interpreting these modalities, recognizing that the BMD value itself is a surrogate marker for bone quality and mechanical competence. The true clinical utility of these assessments is maximized when their results are not viewed in isolation but as inputs for more sophisticated biomechanical models.

This leads to the transformative potential of FEA. FEA has emerged as a powerful tool to bridge the gap between a static BMD measurement and dynamic spinal biomechanics. By integrating patient-specific BMD data derived from QCT with precise three dimensional geometrical reconstructions of the scoliotic spine, FEA allows for a virtual, mechanistic exploration of osteoporosis’s impact. It can quantify how reduced density affects vertebral body strength under load, alters segmental stability at the apex of a curve, and modulates the risk of curve progression. Furthermore, it provides a virtual testing ground for interventions, predicting the biomechanical efficacy of different brace designs or surgical constructs on an osteoporotic spine. This paradigm shift—from correlation to mechanistic simulation—has profoundly deepened our understanding of the “structure-function” relationship in AIS. It offers a plausible biomechanical explanation for the observed clinical heterogeneity: why curves with similar Cobb angles progress at different rates, potentially linked to underlying variations in bone strength and load distribution.

Balancing the promise of this integrated paradigm (BMD + FEA) requires a candid acknowledgment of its current challenges. Most existing FEA models, while insightful, are often simplifications. They may not fully capture the complex, multi-scale nature of bone (trabecular architecture, material anisotropy) or the multi-physics environment (fluid flow in bone marrow, muscle forces). The future research imperative is clear: the development of more sophisticated, multi-scale, and multi-physics coupled simulation models. These advanced models must aim not just for academic fidelity but for clinical translation. The ultimate goal is to evolve FEA from a research tool into a practical, patient-specific CDSS. This requires standardization of modeling protocols, validation against long-term clinical outcomes, and integration into user-friendly software platforms accessible to clinicians.

Clinically, the implications are profound and call for a paradigm shift in management. The evidence compels a recommendation for routine bone health screening in AIS patients. Identifying and managing low BMD should be considered a foundational component of therapy, akin to curve monitoring. Anti-osteoporotic measures, including nutritional optimization (calcium, vitamin D), weight-bearing exercise, and, where indicated, pharmacological agents, could serve as a crucial adjunctive therapy aimed at strengthening the biomechanical substrate. This bone-centric approach must then be seamlessly woven into traditional management. Brace treatment can be optimized by considering the load-bearing capacity of the vertebrae; stiffer braces might be designed or adjusted with the bone’s tolerance in mind. Similarly, surgical planning for spinal fusion must account for bone quality to ensure secure implant fixation and prevent complications like screw pull-out. This holistic approach underscores the necessity of a multidisciplinary collaborative model, involving pediatric orthopedists, endocrinologists, radiologists, and physiotherapists.

In conclusion, the convergence of BMD science and advanced biomechanical modeling is reshaping our comprehension of AIS. It moves beyond viewing the spine merely as a curved column to understanding it as a dynamic, load-adapted structure whose mechanical behavior is intrinsically linked to its material properties. By balancing the robust clinical data from BMD assessments with the mechanistic insights from computational models, we are forging a path toward truly personalized medicine in AIS. The future lies in translating these research insights into integrated clinical workflows that prioritize bone health, leverage predictive biomechanics, and foster interdisciplinary care. The overarching objective remains steadfast: to mitigate curve progression, reduce the need for invasive interventions, and ultimately, enhance the long-term skeletal health and quality of life for individuals living with AIS.
